# Perinatal complications and executive dysfunction in early-onset schizophrenia

**DOI:** 10.1186/s12888-020-02517-z

**Published:** 2020-03-04

**Authors:** Charlotte M. Teigset, Christine Mohn, Bjørn Rishovd Rund

**Affiliations:** 1 Vestre Viken Hospital Trust, Research Department, Wergelands gate 10, 3004 Drammen, Norway; 2 NORMENT Norwegian Centre for Mental Disorders Research, Institute of Clinical Medicine, Postboks 4956 Nydalen, 0424 Oslo, Norway; 3 Department of Psychology, University of Oslo, Postboks 1094 Blindern, 0317 Oslo, Norway

**Keywords:** Early-onset schizophrenia, Executive function, Cognition, Neuropsychology, Obstetric complications, Perinatal complications, Apgar score, Etiological factors

## Abstract

**Background:**

The present study examined the association between perinatal obstetric complications and executive dysfunction in early-onset schizophrenia (EOS), compared to healthy controls. Higher incidences of obstetric complications and more severe executive dysfunctions characterize EOS. Research shows extensive brain maturation in newborns, suggesting them to be particularly vulnerable for perinatal insults. Executive function is mainly mediated by the prefrontal cortex, an area that matures last during pregnancy. Thus, exposure to perinatal complications may influence executive dysfunction in EOS.

**Methods:**

The participants were 19 EOS patients and 54 healthy controls. Executive function was assessed with the D-KEFS Color Word Interference Test and the Wisconsin Card Sorting Test. Information on perinatal obstetric complications and Apgar 5-min scores were obtained from the Norwegian Medical Birth Registry. Associations between perinatal conditions and executive function were studied using stepwise regression analyses.

**Results:**

Perinatal complications, and especially shorter gestational lengths, were significantly associated with significant executive dysfunctions in EOS. Perinatal complications did not affect executive function among healthy controls. A significant relationship between lower Apgar 5-min scores and executive dysfunction was found among both EOS patients and healthy controls.

**Conclusions:**

Exposure to perinatal complications, and particularly a shorter gestational length, was associated with increased executive dysfunction in EOS. Exposed healthy controls did not exhibit similar executive difficulties, suggesting that the EOS patients seemed especially vulnerable for executive deficits due to perinatal insults. The findings indicate that EOS youths learn more slowly and experience more difficulty with problem-solving, which carry important implications for clinical practice. Lower Apgar 5-min scores were associated with executive dysfunction in both groups. Low Apgar score at 5 min may therefore be an important early indicator of executive difficulties among adolescents, independent of diagnosis.

## Background

Obstetric complications (OC) are among the best documented risk factors of schizophrenia [1–5]. A number of pre- and perinatal conditions such as low birth weight [6–9], low gestational length [10–12], fetal hypoxia [13, 14], infections [15–18], maternal stress [19–22] and maternal body mass index (BMI) [23] have been linked to increased risk of schizophrenia, as well as to cognitive deviations [24–27] and structural and functional changes, i.e. in the prefrontal cortex [28–30].

Higher incidences of OC are found among early onset patients [13, 31, 32], and one some suggest the earlier the age of onset, the more likely a history of OC [33]. About 4% of the schizophrenia population develop psychosis between 12 and 18 years of age, also referred to as early-onset schizophrenia (EOS) [34–36]. Compared to later onset, EOS is associated with increased functional and cognitive deficits and more severe developmental and premorbid deviations [35–38]. Research on EOS samples is needed because OC seem to affect subgroups of schizophrenia patients differently [1]. The adolescents are in a period with extensive brain maturation and alterations in cognitive structures and functions [39], which provides the opportunity to explore how disease-related mechanisms may affect facets of neurodevelopment. Such knowledge may contribute to a better understanding of schizophrenia at all ages [40, 41]. Furthermore, being at an early stage of the disease, studies of cognitive changes of EOS patients are not likely to be consequences of long-term pharmacological treatment.

Cognitive deficits, or impairments in cognitive functioning, are a central feature of schizophrenia [42, 43]. Cognitive functions are mental processes that allow us to carry out tasks, and one important cognitive domain is executive function (EF). EFs are higher-level cognitive processes that help us respond in an adaptive manner to the environment; to break out of habits, make decisions and evaluate risks, plan for the future, prioritize actions, and cope with new situations [44, 45]. Major components are inhibition, working memory and shifting [46]. Working memory is also a separate cognitive domain, but shares a common underlying attention component with other parts of EF [47]. The development of EF starts early in life and continues throughout early adulthood [48, 49]. During the preschool years, inhibition improves extensively, while working memory and shifting improves more successively throughout development [46]. The ability to perform more complex tasks comes in early adulthood [50], therefore, examination of EF in EOS needs to include age-matched healthy controls (HC).

Most schizophrenia patients have impairments in EF that are present at illness onset and throughout all stages of the illness [51–58]. Executive dysfunction involves impaired reasoning and problem-solving, reduced emotional regulation, and the inability to use appropriate contextual information to generate and implement adaptive behavior [17, 59, 60]. Whereas good EF is related to treatments success, degree of self-care and occupational functioning, executive dysfunction is associated with functional loss and disabilities [61–63]. It also contributes to other cognitive disabilities [64]. Extensive executive dysfunction has been reported in EOS [54, 65–68], and with more severe deficits compared to later illness onset [69, 70].

Evidence suggests an association between OC and executive dysfunction in schizophrenia [71]. Adult patients exposed to prenatal infections have more profound disabilities than unexposed patients [17]. Research also indicates a link between perinatal, but not prenatal OC, and executive dysfunction [72]. Even though more severe executive deficits occur in EOS, the relationship between OC and EF remains unclear in this group. One study found that fetal exposure to influenza was associated with reduced EF in children who later develop psychoses [73]. In healthy children, executive dysfunction has been associated with prenatal exposure to medication [74] and alcohol [75], as well as to maternal anxiety [76], and some suggest that maternal stress contributes to preterm birth and shorter gestational age [77, 78].

A variety of OC may potentially disturb the fetus’ neural development and cause malformations of the brain [79–81]. Fetal exposure to OC may create a local brain lesion which is reactivated in adolescence when synaptic pruning occurs [82]. Another theory claims that obstetric exposure leads to a continuity of cognitive deviances that cumulatively result in brain pathology and schizophrenia onset [83]. The association between OC and cognitive deficits often appears in schizophrenia patients, but not in HC [1, 24, 25, 80], suggesting a biological vulnerability to develop schizophrenia that interacts with certain complications. The diverse cognitive processes included in EF are mainly mediated by the prefrontal cortex system [28, 29, 54], and neural networks associated with decision making and cognitive control are located in the dorsolateral prefrontal cortex [84]. During pregnancy, the prefrontal cortex is the last to mature, thus, may be more vulnerable to growth abnormalities [85]. However, it seems likely that also other perinatal OC would influence the area in the same way. Structural images of neonates, show considerable brain maturation starting at birth, that potentially represents a window of vulnerability for perinatal insults [86].

A measure that evaluates the overall status of the newborn, as well as the potential consequence of OC, is the Apgar score. The score is used worldwide in the first and fifth minute after birth [87], and is valuable in contemporary practice [88]. The Apgar score seems influenced by a variety of OC [89] and reflects their severity, but without specifying the causes and outcomes [90–92]. Previous results show that exposure to OC and lower Apgar 5-min scores are more frequent in teenagers at risk for psychosis, and is associated with conversion to schizophrenia [93, 94]. Moreover, in the general population, lower Apgar 5-min scores have been linked to severe neurological outcome [95–98], and to cognitive deficits in early adulthood [90] and in adolescence [92], and may also be associated with executive dysfunction.

The present study is part of a research project at the University of Oslo, investigating the relationship between OC and cognition in EOS. In a previous study, we reported that shorter gestational length was associated with increased deficits in generalized cognition and processing speed in EOS [99]. The study included seven cognitive domains. Some of the domains involve problem-solving and working memory, but the study did not examine common measures of EF. Nevertheless, previous findings suggested that perinatal complications and especially gestational length, could have a special impact on EOS patients. Since the prefrontal cortex system is the last to mature, it may be more influenced by such complications. A likely hypothesis is that exposure to perinatal insults affects neural networks in prefrontal areas and disturbs the development of EF more than other cognitive domains. Therefore, in the present study, we included EF tests that are found to be sensitive to frontal lobe deficits [57, 68, 100–106]; the Wisconsin Card Sorting Test (WCST) and the Delis-Kaplan Executive Function System (D-KEFS) Color-Word Interference Test (CWIT) [107, 108]. Both tests are commonly used in adult schizophrenia and are found to be equally sensitive in EOS [54, 64].

Higher incidences of OC and executive dysfunction characterize EOS, but the relationship between the two is still unclear. EF is mainly mediated by the prefrontal cortex, an area that matures last during the pregnancy. In this period, the newborn seems especially vulnerable for perinatal insults [86]. Lower Apgar 5-min scores have been associated with cognitive deficits in adolescence [92]. Thus, the main aim of the present study is to examine the association between perinatal OC and executive dysfunction in EOS, compared to HC. Our first hypothesis is that we expect to find a relationship between perinatal conditions, especially lower birth weight and shorter gestational length, and reduced EF in EOS. Our second hypothesis is to find an association between lower Apgar 5-min scores and executive dysfunction in EOS.

## Methods

### Subjects

This research project is a part of the Early-onset Study at the University of Oslo [39, 99, 109, 110]. The patients were recruited from different inpatient and outpatient units in Oslo and the region of Eastern Norway. Inclusion criteria were age (12 to 18), and diagnosis; broad schizophrenia-spectrum disorder according to DSM-IV (Paranoid schizophrenia: *n* = 2 (11%), Undifferentiated schizophrenia: *n* = 6 (32%), Schizoaffective disorders: *n* = 3 (16%), Residual schizophrenia: *n* = 1 (5%) and Psychosis not otherwise specified (NOS): *n* = 7 (7%)). Patients were excluded if they had a history of central nervous system pathology or trauma (loss of consciousness for greater than 30 min and/or any neurological sequelae), or if estimated with an IQ less than 70. Twenty-one patients (out of a total of 29) gave their written informed consent to the collection of data about OC from The Norwegian Medical Birth Registry (NMBR) [111]. Two patients were not born in Norway and had to be excluded, resulting in a total of 19 patients used for further analysis.

The HC consisted of 67 subjects, of which 54 consented to retrieval of data about OC from the NMBR. The HC were recruited through personal letters to a group of randomly selected individuals from the Norwegian population registry or through advertisements in four schools in Oslo and the region of Eastern Norway. They were matched to patients on gender, age and education. The HC were screened for mental problems using the Mini-International Neuropsychiatric Interview (M.I.N.I.) screening module [112], and a positive response to any of the questions lead to exclusion from the study, as were any known brain injury, neurological disease, or an IQ < 70.

The Wechsler Abbreviated Scale of Intelligence (WASI) was used to measure IQ [113]. A complete description of the study was given all participants, and written informed consent was obtained (also from parents if the participant was younger than 16). The study was approved by the Regional Committee for Medical Research Ethics and the Norwegian Data Inspectorate.

Demographic and clinical characteristics are presented in Table 1.

**Table 1:** Demographic and clinical variables

	EOS (*N* = 19)	HC (*N* = 54)	Group statistics
Gender male/female (%)	9/10 (47.4/52.6)	27/27 (50/50)	X2 0.39, p .84
Age in years (SD)	15.2 (1.9)	15.4 (2.0)	T 0.49, p .63
Hand wright/left (%)	16/3 (84.2/15.8)	47/7 (87/13)	X2 0.09, p .76
Mother’s age at delivery (SD)	28.2 (5.7)	30.7 (4.8)	T 1.70, p .10
Father’s age at delivery (SD)	31.7 (5.9)	32.9 (5.9)	T 0.76, p .45
Mother’s education in years (SD)	13.3 (2.4)	15.4 (2.6)	T 3.29, p .002**
Father’s education in years (SD)	13.5 (2.6)	15.3 (2.6)	T 2.44, p .02*
IQ (SD)	98.9 (13.0)	110.4 (13.4)	T 3.28, p .002**
DUP in weeks, mean (SD)	30.2 (45.5)	–	
GAF function, mean (SD)	51.7 (15.7)	–	
GAF symptom, mean (SD)	52.1 (14.4)	–	
PANSS total score, mean (SD)	55.3 (12.9)	–	

*EOS* Early-onset schizophrenia, *HC* healthy controls. T and X2: Group tests of significance

* = *p* < .05, ** = *p* < .01

### Clinical assessments

The Structured Clinical Interview for DSM-IV (SCID) [114], modules A-D, was used for diagnostic purposes, and the age at onset was defined by the first SCID-verified psychotic episode. Trained clinical researchers carried out diagnostic evaluations and participated in the SCID training program at the University of California, Los Angeles (UCLA) [the mean overall kappa = 0.77 [109]]. Psychiatric symptoms were assessed by the Positive and Negative Syndrome Scale (PANSS) [115], and global functioning was assessed with the Global Assessment of Functioning Scale – split version (GAF) [116].

### Assessment of obstetric complications

Assessments of OC were based on collected data from the NMBR [111]. The registry stores information about all births in Norway, including maternal health before and during pregnancy, and any complications arising during pregnancy or birth, as well as information about medication during pregnancy, labor interventions, birth complications, maternal complications after birth, whether this was a live birth, any diagnoses in the child or evidence of congenital abnormalities. All pregnancies ending after week 12 in Norway are notifiable to the NMBR. Information about the mother’s occupation, smoking and alcohol habits are only registered if the mother consents. The country’s maternity units are responsible for notifying births to the NMBR, and The Ministry of Health and Care Services, pursuant to the NMBR Regulations, requires health authorities to notify births electronically. The aim is to streamline and improve the reporting quality of birth notification. To ensure data quality, the NMBR is routinely linked with the Central Population Register. The Norwegian Institute of Public Health manages the NMBR and is the registry and data controller. Collection and processing of health data in the registry is governed by national regulations [111].

In accordance with our hypotheses, we selected perinatal complications available from the NMBR related to delivery complications, labor interventions, and the newborn’s health. A total of nine perinatal variables were included in the analyses. In addition, we included Apgar 5-min scores. All the complications were analyzed separately (see Table 2).

**Table 2:** Perinatal obstetric complications and Apgar 5-min scores

	EOS (*N* = 19)	HC (*N* = 54)	Group statistics
Gestational length, weeks (SD)	38.8 (3.1)	39.8 (1.7)	T 1.33, p .20
Birth weight, grams (SD)	3304.2 (855.5)	3583.3 (560.5)	T 1.33, p .20
Birth weight < 2500 g	15.8%	3.7%	X^2^ 3.22, p .07
Length in cm (SD)	49.4 (2.9)	50.5 (2.2)	T 1.44, p .16
Dystocia	15.8%	9.2%	X^2^ 0.61, p .43
Bleeding < 500 ml	10.5%	5.6%	X^2^ 0.54, p .46
Use of forceps	5.3%	7.4%	X^2^ 0.10, p .75
Use of vacuum	5.3%	7.4%	X^2^ 0.10, p .75
Emergency ceasarean section	10.5%	7.4%	X^2^ 0.18, p .67
Apgar 5-min, mean (SD)	9.2 (0.8)	9.2 (0.5)	T 0.34, p .74

*EOS* Early-onset schizophrenia, *HC* Healthy controls, T and X2: Group tests of significance

### Neuropsychological assessments

All participants were tested with the Wisconsin Card Sorting Test (WCST) [108], D-KEFS Color Word Interference Test (CWIT) [107]. The WCST and the CWIT are proven to be highly sensitive to executive dysfunction in schizophrenia and are among the most commonly used in assessing EF [17, 54, 64, 105, 117–120].

The WCST assesses the participants capacity for mental flexibility, cognitive inhibition and abstraction. The participants are required to sort a series of cards to key cards that vary in shape, color, and number of shapes. The sorting principles are deduced from feedback provided by the computer, and new principles are presented without warning throughout the trial. As much as ten measures from the computerized version of WCST can be obtained. Studies often only report perseverative responses, and a typical finding is that schizophrenia patients demonstrate a tendency to perseverate in producing an inappropriate response despite negative feedback, which resembles the perseveration often seen in patients with prefrontal cortex damage [106]. However, all measures cover different aspects of EF [118] and are suggested to be important to include in studies of prefrontal cortex deficits and schizophrenia [106]. Hence all measures were included for further analyses: total correct (the total number of correct responses); total errors (the total of all the incorrect responses, should be the same as the sum of perseverative and non-perseverative errors); categories completed (the number of runs of 10 correct responses); perseverative responses (the number of incorrect responses that would have been correct for the preceding category / rule); perseverative errors (the number of errors where the participant has used the same rule for their choice as the previous choice); non-perseverative errors (all the remaining incorrect responses other than the perseverative errors); conceptual level responses (the number of correct responses in runs of three or more, divided by the number of trials × 100; measures insight into sorting principles and indicate conceptual capacity even in individuals who show perseveration); trials to complete first category (the total number of trials needed to achieve the first 10 consecutive correct responses; high scores indicate a poor ability to abstract and generalize); failure to maintain set (the number of times five or more consecutive correct responses occur without completing the category; shows the inability to continue with a strategy that has been successful); and learning to learn (indicates the capacity to profit from one test to another).

The CWIT measures the capacity for verbal inhibition, by assessing the ability to inhibit cognitive interference that occurs when the processing of a specific stimulus feature impedes the simultaneous processing of a second stimulus attribute. It consists of four conditions. First, two baseline conditions measure basic abilities, I: “color naming” of color patches and II: “word reading” color-words printed in black. The two following conditions challenge EF, III: “inhibition” (inhibiting the impulse of reading the words instead of naming the dissonant ink color of the word), and IV: “inhibition/switching” (switching back and forth between naming the dissonant ink colors and reading the words). Two measures (condition III and IV) were used for further analyses; response time measured in seconds (speed), and total errors.

### Statistical analyses

All analyses were carried out using IBM SPSS Statistics version 25. Previous research have investigated EF and reported that the EOS patients performed significantly poorer than their age- and gender matched HC [54], thus group differences in EF will not be of attention in this study.

To analyze associations between perinatal complications and EF, we conducted a series of stepwise linear regression analyses. Each operation included one EF test score as the dependent variable. The independent variable in each operation was one group variable (EOS versus HC), one perinatal OC, and one between-group-interaction variable (diagnosis x OC). All analyses where carried out twice. First with the entire sample (all EOS patients and all HC). Since the control group was considerably larger than the EOS group, all statistical analyses were repeated using pairwise matched samples, based on gender and age, thus with 19 cases in each sample. This procedure did not alter the results, so we report the results of the entire samples. Even though multiple comparisons were carried out, which may lead to Type 1 errors, we did not use Bonferroni adjustments. A problem with such adjustments is that the interpretation of a finding will depend on the number of tests performed. Furthermore, they lead to an increased likelihood of Type II errors [121]. In this study, we aimed to identify relations between OC and executive dysfunction for a future large, national study of adolescents with psychosis. Too conservative statistics would pose the risk of ignoring clinically relevant associations. Thus, adjustments for multiple comparisons were not carried out.

Table 3 shows stepwise regression analyses were the interaction terms were statistically significant (see Table 3 below). Non-significant interaction analyses are not displayed in order to avoid redundancy of data.

**Table 3:** Stepwise regression analyses: The significant main effects and interactions for groups and perinatal complications on executive function, as measured by the Wisconsin Card Sorting Test (WCST) and the D-KEFS Color-Word Interference Test (CWIT)

EF measure	Independent variables	Statistics (*N* = 65–71)	
WCST Trials to complete first category	Group	B 0.20 β 0.23 t 3.90 ***	Adj. R^2^ .24
	OC: Emergency cesarean section	B 0.16 β 0.19 t 1.80	
	Group x OC	B 0.33 β 0.39 t 3.76 *** #	
	Group	B 0.25 β 0.29 t 2.77 **	Adj. R^2^ .25
	OC: Vacuum	B 0.26 β 0.30 t 2.91 **	
	Group x OC	B 0.40 β 0.42 t 3.97 *** #	
	Group	B 0.15 β 0.16 t 1.51	Adj. R^2^ .23
	OC: Gestational length	B − 0.10 β −0.12 t − 0.97	
	Group x OC	B − 0.34 β − 0.47 t − 4.42 *** #	
	Group	B 0.17 β 0.28 t 2.43 *	Adj. R^2^ .15
	OC: Baby’s length	B 0.06 β 0.10 t 0.67	
	Group x OC	B 0.33 β 0.34 t 2.34 * #	
WCST Total errors	Group	B 0.27 β 0.29 t 2.64 *	Adj. R^2^ .19
	OC: Apgar 5-min	B − 0.17 β −0.18 t − 1.44	
	Group x OC	B 0.33 β 0.43 t 3.55 *** #	
WCST Perseverative responses	Group	B 0.28 β 0.29 t 2.65 *	Adj. R^2^ .18
	OC: Apgar 5-min	B − 0.19 β −0.19 t − 1.58	
	Group x OC	B 0.32 β 0.42 t 3.41 *** £	
WCST Perseverative errors	Group	B 0.23 β 0.24 t 2.18 *	Adj. R^2^ .14
	OC: Apgar 5-min	B − 0.13 β −0.13 t − 1.05	
	Group x OC	B 0.30 β 0.39 t 3.09 ** #	
CWIT Inhibition Corrected errors	Group	B 0.15 β 0.15 t 1.32	Adj. R^2^ .10
	OC: Forceps	B 0.12 β 0.12 t 1.02	
	Group x OC	B 0.39 β 0.35 t 3.06 ** #	
CWIT Switching Total errors	Group	B 0.09 β 0.09 t 0.79	Adj. R^2^ .11
	OC: Forceps	B 0.08 β 0.08 t 0.67	
	Group x OC	B 0.37 β 0.33 t 2.84 ** #	
CWIT Switching Corrected errors	Group	B 0.19 β 0.18 t 1.62	Adj. R^2^ .11
	OC: Forceps	B 0.13 β 0.13 t 1.15	
	Group x OC	B 0.39 β 0.35 t 3.07 ** #	

Left column: Dependent variable. Group: EOS vs. controls. OC: perinatal obstetric complication. Group x OC: Interaction term. B: Unstandardized Coefficients Beta, β: Standardized regression coefficient, Adj. R^2^: Adjusted R Square, * *p* < .05, ** *p* < .01, *** *p* < .001, # Interaction effect due to EOS group, £ Interaction effect due to control group

## Results

As displayed in Table 2, we found no significant associations between the two groups (EOS and HC) in exposure to perinatal OC.

In relation to birth weight, dystocia and bleeding, there were no significant differences between exposed and unexposed cases on measures of EF in either groups (data not shown).

When analyzing the EOS group, we found that exposure to several perinatal OC were significantly associated with executive dysfunction (see Table 3). Analyses of associations between perinatal complications and EF measured with the WCST, showed that EOS patients exposed to vacuum and emergency caesarean section during delivery, used significantly more trials to complete the first category of the WCST, than the unexposed cases. Also, in the EOS group, lower gestational length and higher birth length (cm), were significantly associated with more trials to complete first category of the WCST. Lower Apgar 5-min scores in the EOS group significantly increased both the amount of total errors and perseverative errors of the WCST.

Analyses of associations between perinatal complications and EF measured with the CWIT, showed that the use of forceps was significantly associated with inhibitions and switching errors in the EOS group.

In the HC group, analyses of a relationship between perinatal complications and EF as measures by the WCST, showed no significant associations. However, lower Apgar 5-min scores were significantly associated with higher numbers of “perseverative responses”. There were no significant associations between perinatal complications or Apgar 5-min scores and executive dysfunction as measured with the CWIT among HC.

All significant results; main effects and interactions for groups and perinatal complications on EF are presented in Table 3.

## Discussion

Previous results have revealed executive dysfunctions in EOS compared to HC [54]. Moreover, gestational length has been associated with general cognitive deficits in EOS [99].

The present study found no significant association between birth weight, dystocia or bleeding and EF in EOS. However, a variety of perinatal complications seemed linked to reduced EF in EOS. In relation to our first hypothesis, a shorter gestational length in the EOS group was significantly associated with more trials to complete the first category of the WCST. A reduced ability to complete the first category can be explained by a diminished capacity to generate or apply cognitive inhibition. This is often manifested as cognitive control deficits and frequent distraction by non-pertinent inhibition [118]. Hence, the difficulties seem closely related to several clinical symptoms frequently encountered by this illness, such as incoherent thought and speech [122].

Low gestational age has been found to increase the risk for developing schizophrenia [11, 12, 123]. While these studies often concern very preterm birth, the mean length of gestation in our EOS group was not particularly short. The results suggest that length of gestation is especially critical in this group, and that even small reductions in lengths influence neurodevelopment. A possibility is that shorter gestation halts a natural in utero maturation of the prefrontal cortex system, and thereby affects the development of neural networks associated with EF. This hypothesis is in line with research indicating that perinatal complications disturb the extensive brain maturation in newborns, starting at birth [86]. Thus, full term pregnancies seem especially important in this group and may prevent executive dysfunction. Interestingly, we found a small association between the babies’ length at birth and needing more trials to complete the first category of the WCST. This means that the babies who were longer at birth and later develop schizophrenia, had larger executive deficits. This result seems illogical and may be due to type I error. Furthermore, we found no association between executive dysfunction and lower birth weight; a measure often linked to growth abnormalities and poorer neurodevelopment [25, 85]. Though prematurity is often found with growth abnormalities and low birth weight, there is not necessarily a link between the two. The newborn can be premature and still have an average birth weight, and vice versa. However, our results suggest that full term delivery more than birth weight affects the development of EF.

Our findings indicated that EOS cases exposed to emergency caesarean section and the use of vacuum needed more trials to succeed at the first category in the WCST than unaffected cases and controls. Furthermore, those exposed to forceps committed more inhibition and switching errors on the CWIT. The results are based on few participants and should be interpreted with caution. Yet, similar results were reported by Yurgelun-Todd and Kinney [72], who found more profound executive deficits in adults with schizophrenia exposed to perinatal OC. Our findings suggest that these perinatal insults may have a specific impact on EOS and the development of EF in this group. Due to a small sample size, the findings should be replicated in larger samples to ensure reliability. These perinatal insults, as well as gestational length, were mainly connected to the use of more trials to succeed at the WCST, which indicate that EOS patients with a history of perinatal complications learn more slowly and need considerably more practice trials to complete tasks than HC and those with no history of perinatal OC. These patients seem to have a lower learning curve and larger difficulties with problem-solving, which are important implications for clinical practice.

As regards our second hypothesis, in the EOS group, we found that a lower Apgar score at 5-min was significantly associated with more total errors and more perseverative errors in the WCST. However, one measure of executive dysfunction (perseverative responses) was linked with lower Apgar 5-min scores also among HC. As noted earlier, a relationship was found in the general population between lower 5-min Apgar scores and increased risk of severe neurological outcome [95–98]. A Danish study that include almost 20,000 men, showed that lower 5-min Apgar scores were related to neurological disabilities and lower cognitive function in early adulthood (median 19 years) [90]. Similar results from Sweden showed an association between a low Apgar score at 5 min and poor cognitive functioning in adolescence (15–16 years) [92]. Our findings support these conclusions and suggest that Apgar 5-min scores are also associated with executive dysfunction in adolescents, independently of diagnosis. Thus, the 5 min score may be important for identifying executive deficits in youths.

Our results suggested that HC exposed to the same perinatal conditions as the EOS patients, did not exhibit similar executive difficulties. One study found executive dysfunctions in all participants exposed to pre- and perinatal OC (schizophrenia patients, their siblings and HC) [72], however, most research on the relationship between OC and cognition, reports deficits among OC exposed schizophrenia patients, but not among controls with the same OC history [1, 24, 80, 124]. Differences in influence of OC may be explained by unknown genetic or epigenetic factors; suggesting that genetic vulnerabilities to schizophrenia interact with certain complications to cause neurocognitive dysfunctions among those otherwise disposed for psychosis, but not among others [1]. Preti and Wilson [80] support these conclusions and claim that a wide range of genes can positively or negatively influence the effect of different OC. Moreover, the same genes are involved in neurodevelopment. A connection between genes, schizophrenia, neurodevelopment and neurodegeneration is also reported in other studies [125, 126]. Recent evidence shows that a sizable fraction of genes in the schizophrenia GWAS (genome-wide association studies), directly influence placental biology and health and can predict complicated pregnancies [127]. Shorter gestational lengths may therefore affect those vulnerable to psychosis, but genetic vulnerability in the fetus may also affect the length of gestation.

It is important to note that a variety of conditions may inflict on perinatal complications. Individual maternal characteristics, i.e. BMI [128], stress [129, 130] and exercise [131] have been linked to alterations in immune functioning, susceptibility to infection, and schizophrenia [132]. Such conditions most likely affect the pregnancy and may influence prematurity or vulnerability to perinatal insult. Such characteristics were not available to us but could have had an impact on our findings. Nonetheless, research that includes a variety of potential interactions is important for future investigations of a relationship between OC, schizophrenia and neurodevelopment.

Finally, our findings indicated that the WCST is particularly sensitive to executive dysfunctions, which is consistent with earlier reports [57, 104], especially in the EOS group [64]. An explanation may be that inhibition is the first EF to mature, while working memory and shifting show a more gradual improvement throughout development [46]. The WCST mainly assesses inhibition deficits and may therefore give a better assessment of executive dysfunction in the adolescent group. We found stronger associations between perinatal conditions and executive deficits, than between OC and other cognitive deficits in the EOS group reported in our previous study [99]. Perinatal insults could therefore affect EF more profoundly than other cognitive domains in this group. A possible explanation is that perinatal complications disturb neural networks mainly located in the prefrontal cortex, causing executive dysfunction in EOS. It may also be that the dysfunction indirectly affect other cognitive abilities as a result of inadequate strategies to approach, plan or accomplish cognitive tasks [133]. Hence, the deficits in the other cognitive domains are diffused and appear less prominent. Larger samples with more statistical power might reveal stronger evidence of such associations in EOS.

A major strength of this study are the comprehensive cognitive assessments of EOS youths coupled with data on perinatal OC retrieved from the NMBR. Most research on OC and schizophrenia use obstetric information collected from mothers based on their recollection, which may be unreliable [93, 134]. Many studies investigate EOS in comparison to other schizophrenia patients or other diagnosis groups. But our use of a healthy control group offers a unique opportunity to examine how perinatal insults influenced EF in EOS patients compared to HC.

Major limitations apply to this study. Firstly, our sample size is small. This limits the ability to generalize our findings to a wider context of neurodevelopment among schizophrenia patients. There may be covariates we have not considered, and some of the null results may be explained by Type II errors. Our findings are therefore preliminary and should be replicated in larger samples. Secondly, the study included many perinatal measurements and multiple tests were conducted without adjusting for multiple comparisons. This increases the risk of a Type I errors. Finally, the sampling area for all participants was eastern Norway, which may imply regional influences on the results. However, even with such a small sample, our results exhibited clear, significant associations between perinatal OC and executive dysfunction, and this strengthens our findings. Moreover, our sample size is not particularly smaller than those of other studies in this field [17, 24–26]. Even so, future studies with larger samples are necessary.

## Conclusion

Our findings indicate that perinatal complications are associated with impaired EF in EOS. HC exposed to the same perinatal OC, did not exhibit the same deficits, indicating that EOS patients seem particularly vulnerable for executive dysfunction due to perinatal obstetric events. These patients seem to learn more slowly and experience more difficulty with problem-solving, which carry important clinical implications. Finally, our data reveal that lower Apgar 5-min scores are associated with reduced EF, both in the EOS group and among HC. Low Apgar at 5 min may therefore be an important early indicator of executive dysfunction among adolescents, independent of diagnosis.

## Data Availability

The data that support the findings of this study are available from Professor Bjørn Rishovd Rund but restrictions apply to the availability of these data, which were used under license for the current study, and so are not publicly available. Data are however available from the authors upon reasonable request and with permission of Bjørn Rishovd Rund and will on request be presented anonymously in relation to diagnosis, age and gender.

## Abbreviations

BMI: Body Maze Index
CWIT: D-KEFS Color Word Interference Test
EF: Executive Function
EOS: Early-onset Schizophrenia
GAF: The Global Assessment of Functioning Scale – split version
HC: Healthy Controls
M.I.N.I: Mini-International Neuropsychiatric Interview
NMBR: The Norwegian Medical Birth registry
OC: Obstetric Complications
PANSS: The Positive and Negative Syndrome Scale (PANSS)
SCID: The Structured Clinical Interview for DSM-IV
UCLA: The University of California, Los Angeles
WASI: Wechsler Abbreviated Scale of Intelligence
WCST: Wisconsin Card Sorting Test
